# Biomass and enzymatic activities of marine bacteria in the presence of multiple metals

**DOI:** 10.1007/s42770-023-00993-5

**Published:** 2023-05-22

**Authors:** J. A. P. Bitencourt, L. P. T. Chequer, C. C. Waite, G. Oliveira, A. M. S. Oliveira, D. C. Pereira, M. A. C. Crapez

**Affiliations:** 1 Instituto Tecnológico Vale, Belém, PA CEP 66055-090 Brazil; 2 Departamento de Biologia Marinha, Programa de Pós-Graduação Em Biologia Marinha E Ambientes Costeiros, Universidade Federal Fluminense, Niterói, RJ CEP 24020-150 Brazil; 3 School of Earth and Environmental Sciences, University of Queensland, St. Lucia, Brisbane, QLD 4072 Australia

**Keywords:** Bioremediation, Metals, Esterase enzymes, Dehydrogenase enzymes

## Abstract

Marine environments are a repository for metals, and humans have enhanced this phenomenon over the years. Heavy metals are notoriously toxic due to their ability to biomagnify in the food chain and interact with cellular components. Nevertheless, some bacteria have physiological mechanisms that enable them to survive in impacted environments. This characteristic makes them important as biotechnological tools for environmental remediation. Thus, we isolated a bacterial consortium in Guanabara Bay (Brazil), a place with a long metal pollution history. To test the growth efficiency of this consortium in Cu–Zn-Pb-Ni–Cd medium, we measured the activity of key enzymes of microbial activity (esterases and dehydrogenase) under acidic (4.0) and neutral pH conditions, as well as the number of living cells, biopolymer production, and changes in microbial composition during metal exposure. Additionally, we calculated the predicted physiology based on microbial taxonomy. During the assay, a slight modification in bacterial composition was observed, with low abundance changes and little production of carbohydrates. *Oceanobacillus* *chironomi*, *Halolactibacillus miurensis*, and *Alkaliphilus oremlandii* were predominant in pH 7, despite *O. chironomi* and *Tissierella creatinophila* in pH 4, and *T. creatinophila* in Cu–Zn-Pb-Ni–Cd treatment. The metabolism represented by esterases and dehydrogenase enzymes suggested bacterial investment in esterases to capture nutrients and meet the energy demand in an environment with metal stress. Their metabolism potentially shifted to chemoheterotrophy and recycling nitrogenous compounds. Moreover, concomitantly, bacteria produced more lipids and proteins, suggesting extracellular polymeric substance production and growth in a metal-stressed environment. The isolated consortium showed promise for bioremediation of multimetal contamination and could be a valuable tool in future bioremediation programs.

**Supplementary Information:**

The online version contains supplementary material available at 10.1007/s42770-023-00993-5.

## Introduction

Metals are considered a severe environmental threat due to their toxicity, nondegradability, persistence, and ability to bioaccumulate through the food chain [1]. Increased concentrations of metals, including micronutrients, can be toxic to biota [1]. Large amounts of metal waste are annually released into the sea from industrial discharge, highway runoff, domestic sludge, and harbor activities.

The environmental behavior of metals is strongly dependent on their chemical form, which influences mobility, bioavailability, and toxicity to organisms. Metal speciation can occur in marine sediments, influencing their bioavailability and, therefore, metal biomagnification. Metal speciation results in highly complicated remediation concerns in water body systems [2].

Some reports show that metals in sediments inhibit microbial activities [3, 4] due to competitive adsorption with micronutrients, affecting enzymatic activities, influencing bacterial abundance [5, 6], and reducing bacterial diversity [7]. Multimetal have inhibitory effects, i.e., homeostasis disturbances in microorganisms, quantified by means of biomarkers such as dehydrogenase (DHA) and esterases (EST), which show a reduction in energy generation and an increase in energy demand, respectively [8-12]. Despite the deleterious nature of multimetal pollution events, they do not affect certain microbial populations [12, 13], suggesting that these populations could be used as a potential tool for metal bioremediation.

Bioremediation can employ microorganisms and is considered an attractive, eco-friendly, and low-carbon-footprint alternative [14] to conventional physical and chemical methods. This process uses microorganisms as biosorbents (energy independent) [15, 16] or bacterial metabolism (energy dependent) that transform toxic heavy metals into less harmful products [17, 18] to reclaim polluted environments [18]. It is a viable technology to eliminate or chemically transform metals and metalloids [16]. The microbial world has high metabolic and physiological diversity, enabling microbes to sense metal bioavailability and counter their toxicity [17, 18].

This preliminary work aims to isolate microbial consortia capable of growth in the most common multimetal exposure conditions in marine environments (Cu–Zn-Pb-Ni–Cd). For this study, we sought out a location with a long multimetal pollution history. We tested and measured the activity of key enzymes of microbial activity under acidic (4.0) and neutral pH conditions and followed changes in the populations during metal exposure. The consortium’s ability to grow in contact with the studied multiple metals indicates its potential as a candidate for bioremediation in the future.

## Methods

### Isolation of bacterial consortia and bioassays

Surface sediment samples collected in Guanabara Bay in the Jurujuba Sound area (22°56ʹ5ʺ S, 43°6ʹ44ʺ W), Rio de Janeiro, Brazil, were placed in a sterile saline medium at a 1:10 ratio and incubated for 10 days at room temperature. Next, aliquots (1:10) were removed, seeded in a liquid medium containing 5 g.L^−1^ yeast extract and 5 g.L^−1^ urea in 250 mL Erlenmeyer flasks and incubated at room temperature for 7 days [12]. Heavy metals were also added to these culture media. Standard solutions of Cu (CuSO_4_.5H_2_O), Zn (ZnSO_4_.7H_2_O), Pb [Pb(NO_3_)_2_], Ni (NiSO_4_.6H_2_O), and Cd [Cd(NO_3_)] were prepared at a concentration of 10,000 µg.mL^−1^, and the final concentrations added to the culture media were 7.8 µg.L^−1^ Cu; 0.12 mg.L^−1^ Zn; 0.21 mg.L^−1^ Pb; 74 µg.L^−1^ Ni; and 0.04 mg.L^−1^ Cd, according to the environmental quality standard limit of CONAMA Resolution 357/2005 [19], class 2 for saline waters. The culture media were autoclaved for 20 min at 120 °C. The flasks that showed biomass growth in the presence of the Cu–Zn-Pb-Ni–Cd mixture were selected, and the cultures were kept in the same liquid culture medium used for isolation.

The bioassays were performed in triplicate in a liquid medium containing 5 g.L^−1^ yeast extract; 5 g.L^−1^ urea; 7.8 µg.L^−1^ Cu; 0.12 mg.L^−1^ Zn; 0.21 mg.L^−1^ Pb; 74 µg.L^−1^ Ni; and 0.04 mg.L^−1^ Cd and incubated at room temperature. The pH of the culture media was adjusted to 4.0 and 7.0, and the samples were autoclaved for 20 min at 120 °C. The bioassays included a control (without metals) and the treatment group (with multimetal solution) and lasted 11 days. Analyses were performed on days 0, 5, and 11 (T0, T5, and T11).

### Cell number quantification

Two milliliters of culture medium was filtered through a sterile 0.22 μm Millipore membrane and stained with acridine orange. Cells were counted under an epifluorescence microscope at 1000 × magnification (Axioskop 1, Zeiss, Texas Red triple filter – DAPI – fluorescein isothiocyanate) according to Kepner et al. [20].

### DNA extraction, amplification, and microbial identification

Total DNA was extracted from 1 mL samples using the UltraClean Microbial DNA Isolation Kit (MOBIO). Library construction was performed using the Illumina 16S Metagenomic Library (Illumina, San Diego, CA, USA) with the V3 and V4 regions of the 16S rRNA gene using the universal primers SD-Bact-0341-bS-17-N and SD-Bact-0785-aA-21-N [21]. Amplification was performed in a thermocycler with the following temperature profile: initial denaturation at 95 °C for 3 min, followed by 25 cycles of 95 °C, 63 °C for 30 s, 57 °C for 30 s, and 72 °C for 30 s, and final extension at 72 °C for 5 min.

DNA quantification was performed using the Qubit™ dsDNA HS (High Sensitivity) Assay (Thermo Fisher Scientific). The quality of the amplicons was evaluated according to fragment size using capillary electrophoresis with an Agilent Technology 2100 Bioanalyzer.

According to the manufacturer’s instructions, amplicons were purified using the Agencourt AMPure XP Magnetic Bead Kit (Beckman Coulter, Inc., Brea, USA). Then, single-line adapters (indexes/barcodes) were added to each sample in the PCR index step using the indexes from the Nextera XT Library Preparation Kit (Illumina, San Diego, CA, USA).

Subsequently, the libraries were standardized to a concentration of 4 nM, and the genome pool was prepared according to the Illumina 16S Metagenomic Library (Illumina, San Diego, CA, USA) preparation protocol. The sequencing run was performed on the Illumina MiSeq platform using the MiSeq V3 600 cycle run kit.

The identification of microbial communities was performed in PIMBA [22], a pipeline based on the Quantitative Insights Into Microbial Ecology (QIIME) pipeline [23]. Then, trimming and quality filtering steps (Phred > 20) were performed in Prinseq [24]. The forward and reverse sequences were assembled using the Pear assembler [25]. After assembly, the duplicated sequences were removed, followed by data selection according to sequence abundance (considering a count of at least two). To improve the quality of the metabarcoding, all sequences smaller than 297 bp were filtered, and erroneous operating taxonomic units (OTUs) were removed by the LULU algorithm [26]. Next, sequences with > 97% similarity (90% coverage and 95% identity) were grouped into OTUs using USEARCH 7 (https://www.drive5.com/usearch/). The taxonomic attribution of each OTU was performed by comparison with sequences available in the Ribosomal Database Project (RDP) database (https://rdp.cme.msu.edu) [27]. All R analyses and graphs based on OTUs were generated using the Phytools package (Phylogenetic Tools for Comparative Biology, http://www.phytools.org).

FAPROTAX was used to predict the potential functions of the bacterial community that formed the consortia in the control, pH 4‒7, and multimetal exposure groups. FAPROTAX was constructed to identify metabolically or otherwise ecologically relevant prokaryotes and integrate multiple culturable bacteria whose primary functions have been reported in the literature [28]. The heatmap was generated in R using the Pheatmap package and the Ward D2 cluster distance [29].

### Quantification of enzyme activities

Dehydrogenase activity (DHA) was measured by the reduction of iodonitrotetrazolium chloride (INT) to formazan INT. INT acts specifically as an artificial electron acceptor when the succinate dehydrogenase complex in the electron transport chain is reoxidized. DHA was evaluated in triplicate according to Stubberfield et al. [8]. Aliquots of 1 mL were taken from each culture medium, and 0.2 mL of 8 mM INT was added. After shaking, the tubes were incubated in the dark. Readings were performed at 458 nm, and the activity was expressed in μL O_2_.h^−1^.mL^−1^.

Esterase (EST) activity was analyzed according to Stubberfield et al. [8]. The analysis is based on a fluorogenic compound (fluorescein diacetate, FDA), which is enzymatically transformed into fluorescent products that can be quantified by spectrophotometric assay with incubation of the samples at 24 °C for 75 min. Readings were performed at 490 nm, and the results are expressed in μg fluorescein.h^−1^.mL^−1^. These extracellular enzymes act on biopolymers and transform them into low-molecular-weight organic carbon. This method is based on the hydrolysis of fluorescein diacetate to fluorescein.

### Quantification of biopolymers

The concentrations of total biopolymers (carbohydrates (CHO), lipids (LPD), and proteins (PRT)) were determined in triplicate. CHO was quantified according to DuBois et al. [30], using glucose as a standard. LPD was extracted with chloroform and methanol and analyzed according to Marsh et al. [31], and tripalmitin was used as the standard. PRT was determined according to Hartree [32], and bovine albumin fraction V (Sigma) was used as the standard.

### Data analysis

The bacterial range of species richness and diversity was assessed. Richness was calculated using the number of taxa, the Shannon‒Wiener index was used to evaluate the bacterial diversity, and Simpson’s index was used to estimate the dominance of taxa. The Wilcoxon test was used to compare the bacterial richness and Shannon and Simpson indexes between the control and treatment groups. The diversity was calculated using R Phytools and microbiomeSeq (an R package for microbial community analysis in an environmental context—https://github.com/umerijaz/microbiomeSeq).

The effects of the pH on bacterial activities in the control and treatment at different times (T0, T5, and T11) were evaluated using three-level nested analysis of variance (nested-ANOVA). Tukey’s pairwise post hoc tests were used to confirm the significance of the nested-ANOVA results. All statistical analyses were calculated with a 95% confidence interval (*p* < 0.05) using the R statistical environment [33].

## Results and discussion

Analysis of variance (nested-ANOVA) indicated that the interactions between the factors pH (4.0 and 7.0), bioassays (control and treatment), and times (T0, T5, and T11) were significant for most of the analyses performed in the experiment (Online Resource 1).

**Fig. 1:**
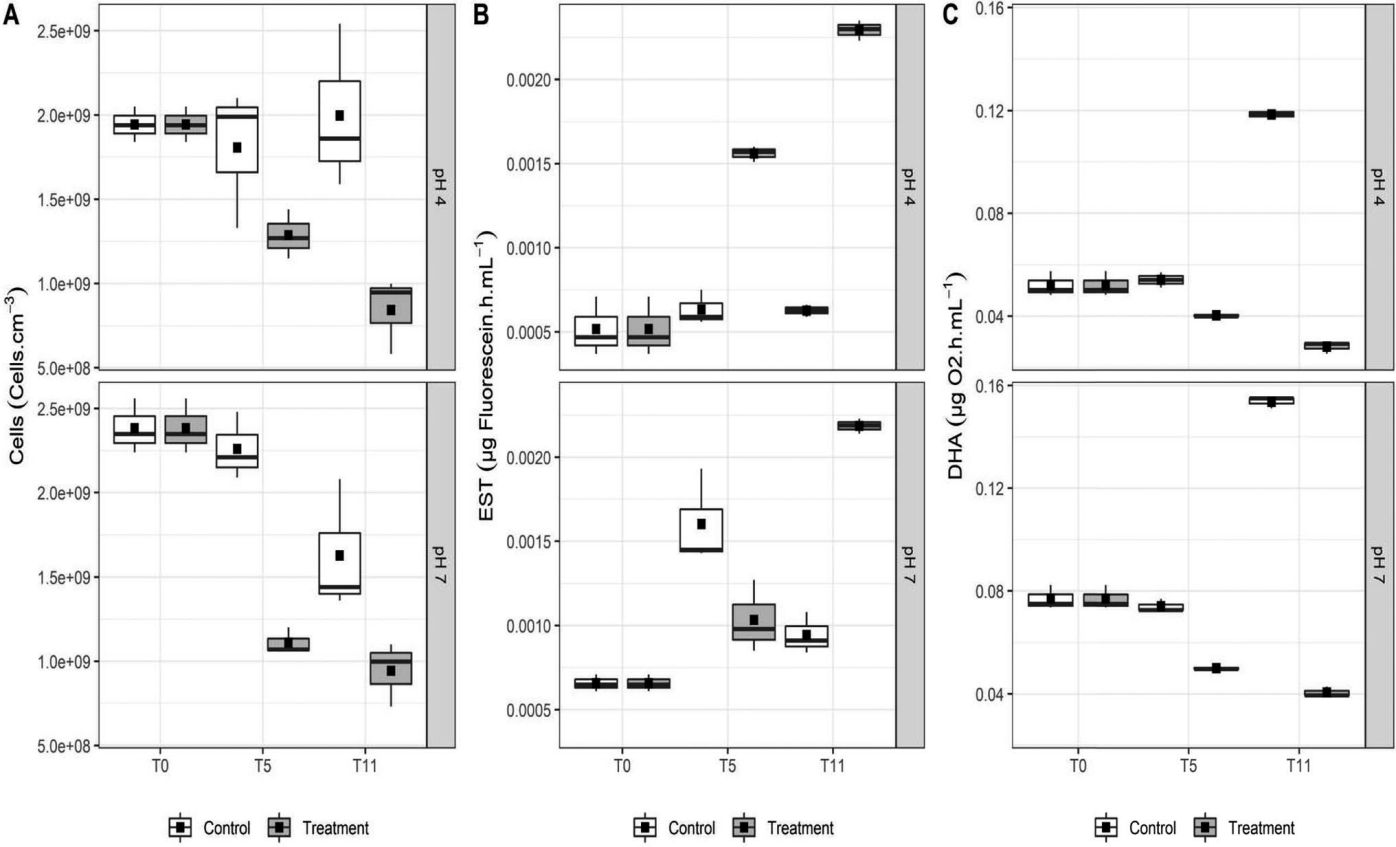
Growth and enzyme production of tested bacterial consortium in the presence of metal solution (Cu, Zn, Pb, Ni, and Cd) at concentrations of 0 (control) and environmental standard limit (CONAMA Resolution 357/2005) and adjusted pH (4 and 7) for 11 days (T0, T5, and T11). **A** Number of cells (cell.mL^−1^). **B** Esterase (EST) (μg Fluorescein.h. mL^−1^). **C** Dehydrogenase (DHA) (µg O2.h.mL^−1^) activities

The number of cells remained at 10^9^ cells.cm^−3^ (Fig. 1A) in the controls and up to T5 in the presence of the multimetal Cu–Zn-Pb-Ni–Cd mixture at pH 4.0 and 7.0. The values were significantly different between the control and the following multimetal treatments (T5 and T11): 7.8 µg.L^−1^ Cu; 0.12 mg.L^−1^ Zn; 0.21 mg.L^−1^ Pb; 74 µg.L^−1^ Ni; and 0.04 mg.L^−1^ Cd (*p* < 0.05). The isolated bacterial consortium maintained high biomass for 11 days of assay, although metals, mainly Cd and Pb, inhibit cell division and the transcription process in bacterial cells and denature nucleic acids and proteins, as reported by Thomas and Benov, and Fashola and colleagues [34, 35].

ESTs catalyze the hydrolysis of the ester linkage of biopolymers greater than 600 Da [9]. This effect is correlated with ATP production, thymidine incorporation, and cell metabolic activities, which participate in the cycling of carbon and nutrient sources [36]. Energy generation (adenosine triphosphate, ATP) was evaluated through DHA [8]. The results for EST in the control at T0 and in the multimetal treatments at T0, T5, and T11 were significantly different at pH 4.0 and 7.0 (*p* < 0.05). As the number of cells decreased, the EST activity increased until reaching ≥ 0.0022 µg fluorescein.h^−1^.mL^−1^ in the treatments at pH 4.0 and 7.0 without multimetal exposure (Fig. 1B). The DHA results for the control at T0 and for the multimetal treatments at T0, T5, and T11 were significantly different at pH 4.0 and 7.0 (*p* < 0.05). After 11 days, the controls had increases in DHA activity of 0.118 μL O_2_.h^−1^.mL^−1^ and 0.154 μL O_2_.h^−1^.mL^−1^ at pH 4.0 and 7.0, respectively. In the same period, the multimetal treatments showed a significant decrease in DHA activity at both pH 4.0 and pH 7.0 (Fig. 1C). The mobility of a heavy metal depends on its ionic radius. Thus, Cu, Zn, Cd, and Pb ions, with smaller ionic radii, have greater mobility at pH > 6.0, while Ni, with a larger ionic radius, has a smaller mobility [37]. Regardless of ionic radius and pH, the metals were able to significantly decrease the enzymatic activity (*p* < 0.05). Similar results were also obtained in the presence of Cu and Cu + Pb [38] and in the presence of Cd [39].

Under disturbance of homeostasis with multimetals, biomarkers such as DHA and EST indicate a reduction in energy generation and an increase in energy demand, respectively [8, 10, 11, 36, 38]. DHA and EST indicated that consortium was stressed out by the presence of multimetals, which increases their need for food and energy to complete their life cycle [40]. It is well known that Cu, Zn, and Cd bind in enzymes involved in energy synthesis [38, 39], making energy production challenging for bacteria. In our bioassay, intracellular carbon supply for energy production continued throughout the bioassay since the activity of the EST was connected to biopolymer degradation processes [8], followed by the activity of the dehydrogenases.

DHA and EST activities have been employed in several studies as biomakers of the toxicity and bioavailability of metals, pesticides, and petroleum hydrocarbons in soil and sediment samples [41-47]. In the presence of stressors, the activity of the EST and DHA in a multimetal exposure was able to sustain 10^9^ cells.mL^−1^, which is expressive biomass.

**Fig. 2:**
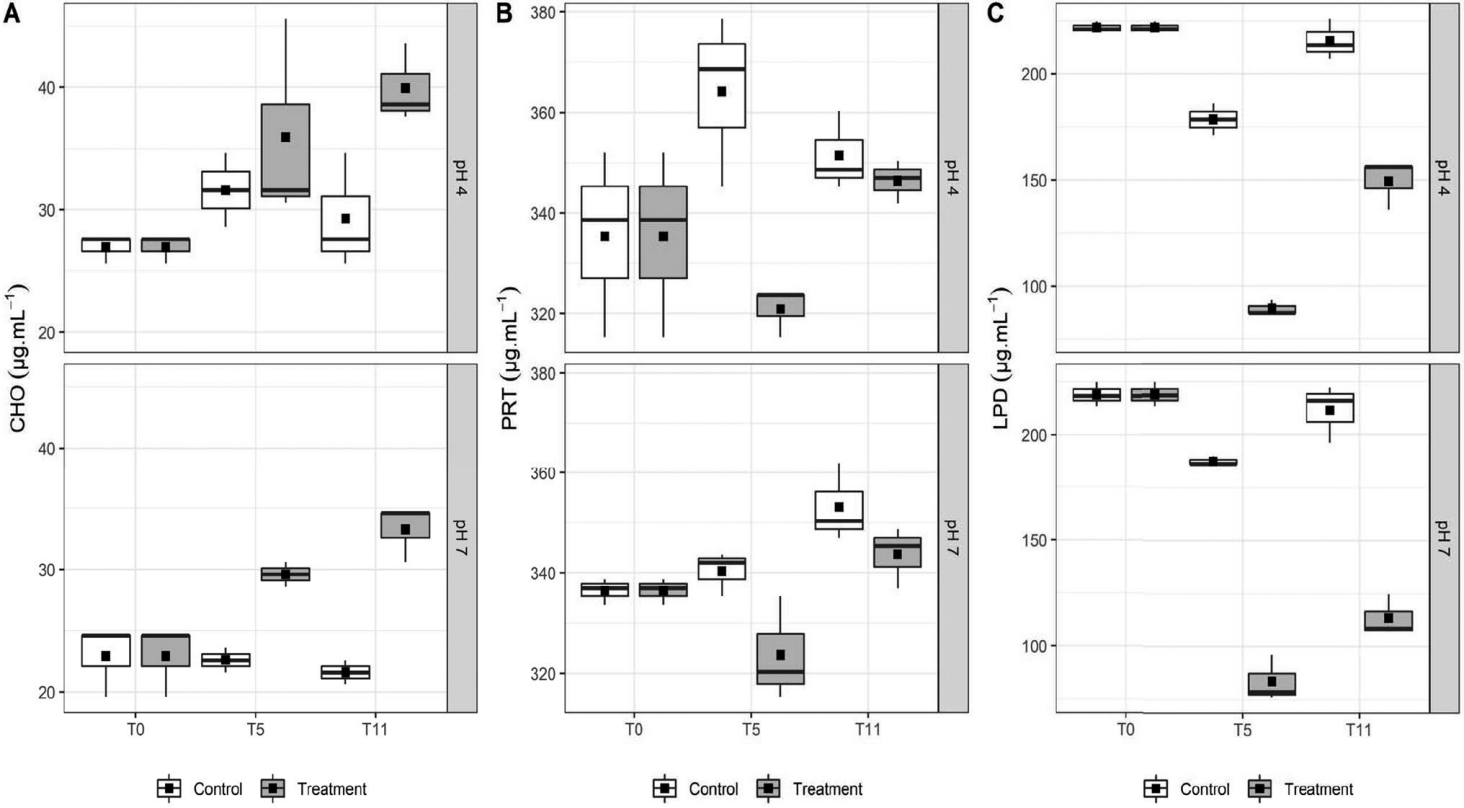
Biopolymeric compounds (µg.mL^−1^) produced by tested bacterial consortium in the presence of metals metal solution (Cu, Zn, Pb, Ni, and Cd) at concentrations of 0 (control) and environmental standard limit (CONAMA Resolution 357/2005) at adjusted pH (4 and 7) for 11 days (T0, T5, and T11). **A** Carbohydrates. **B** Proteins. **C** Lipids

Biopolymer CHO and PRT showed no significant differences between the controls and treatments at pH 4.0 and 7.0 (*p* > 0.05). CHO was not consumed or produced in the presence of coexisting Cu, Zn, Pb, Zn, and Cd (Fig. 2A). The concentrations of PRT in the controls and treatments at pH 4.0 and 7.0 (Fig. 2B), were higher in T11 than in T0, indicating PRT synthesis at the end of assay, suggesting the investment of bacteria in cell maintenance and/or extracellular polymeric substance (EPS) production. In the presence of Cu + Pb, the EPS of *Pseudomonas stutzeri* W228 absorbed 50 µg.mL^−1^ Pb but left Cu (50 µg.mL^−1^) in the liquid culture medium [38]. The EPS from *Oceanobacillus depthus* KBZ 3–2 sequestered both Pb and Zn due to the presence of ionizable functional groups, such as carboxyl, sulfate, and phosphate groups, in proteins and polysaccharides [48]. The EPS of *Bacillus* sp. was also able to sequester Pb, Cu, and Cd [49]. The LPD results for the control at T0 and for the treatments at T0, T5, and T11 were significantly different at pH 4.0 and 7.0 (*p* < 0.05) (Fig. 2C). At these pH values, LPDs were consumed in the controls and with greater intensity in the treatments. However, at T11, LPDs increased, possibly to contribute to the synthesis of EPS.

**Fig. 3:**
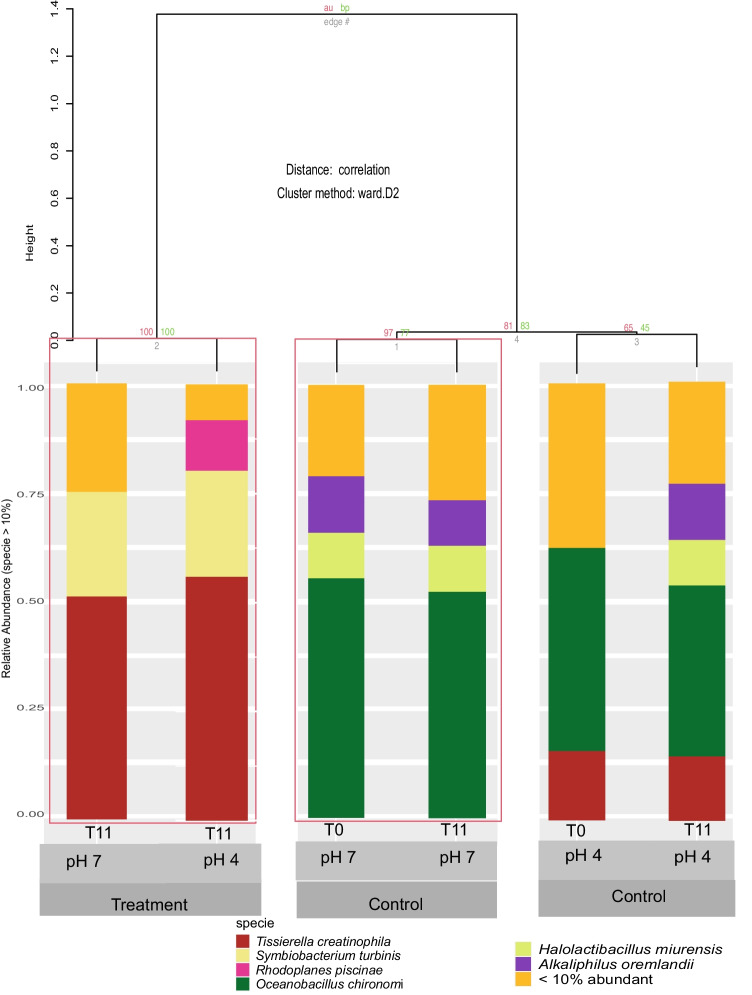
Composition of tested bacterial consortium in the presence of metals metal solution (Cu, Zn, Pb, Ni, and Cd) at concentrations of 0 (control) and environmental standard limit (CONAMA Resolution 357/2005) at adjusted pH (4 and 7) for 11 days (T0, T5, and T11). Nonmetric dimensional scaling (NMDS) analysis was used to show composition clustering according to the treatment

In the nonmetric dimensional scaling (NMDS) analysis, the composition of the bacterial consortium was grouped according to the type of treatment (Fig. 3). At pH 7.0, in the control, *Oceanobacillus chironomi*, *Halolactibacillus miurensis*, and *Alkaliphilus oremlandii* predominated. However, at pH 4.0, the composition of the bacterial consortium changed: *O. chironomi* and *Tissierella creatinophila* were maintained from the beginning, while *H. miurensis* and *A. oremlandii* increased at the end of the experiment (Fig. 3).

The treatment with coexisting Cu–Zn-Pb-Ni–Cd at T11 showed selection in the bacterial consortia at both pH 4.0 and pH 7.0: *T. creatinophila, Symbiobacterium turbinis*, and *Rhodoplanes piscinae* predominated in the presence of multiple metals, replacing *O. chironomi*, *H. miurensis*, and *A. oremlandii*, which were present in the control bioassays (Fig. 3). Only *T. creatinophila* was able to grow in both the control bioassay and in the presence of multiple metals.

*T. creatinophila*, a predominant species in the treatment with Cu–Zn-Pb-Ni–Cd (Fig. 3), was isolated from an area contaminated by sewage in Guanabara Bay, Brazil, and grown in aerobic conditions using yeast and urea extract as carbon and energy sources. According to Harms et al. [50], *T. creatinophila* is strictly anaerobic; the species in that study was also isolated from sewage sludge, used creatine as a source of carbon and energy, and was dependent on selenium.

*O. chironomi* was isolated from freshwater insect eggs, is obligatorily aerobic and optionally alkaliphilic, does not ferment carbohydrates, and grows at pH values ranging from 6.5 to 10, and the predominant fatty acid in the species is anteiso-C_15:0_ [51]. Sami et al. [52] observed *O. chironomi* in the smokeless tobacco “Toombak”, which has trace metals such as chromium, cobalt, and copper. The presence of multiple metals did not favor the growth of *O. chironomi* (Fig. 3), although it was isolated from sediment contaminated by metals [53].

*H. miurensis* grows at an optimal pH of 9.5 and exhibits optimal growth at 37–40 °C. Lactic acid is the primary product of glucose fermentation, and 40–50% of produced lactic acid is converted to formic acid, acetate, and ethanol [54]. *H. miurensis* EPS has 56.1% carbohydrates, and the main monosaccharides are galactose and glucose. These EPS have antioxidant activity against hydroxyls and free radicals [55]. *H. miurensis* grew only in the absence of the Cu–Zn-Pb-Ni–Cd (Fig. 3) and used yeast extract and urea as growth substrates. The EPS synthesized by *H. miurensis* was not able to sequester metals in experiments, in contrast to *Nitratireductor* spp. and *Pseudomonas* spp., which sequestered Zn(II) and Cu(II) at 50 mg/L [56].

*A. oremlandii* is a mesophilic microorganism that was isolated in the presence of lactate and arsenate and grows at pH 8.4. It can ferment lactate, fructose, and glycerol and uses thiosulfate as the final electron acceptor with acetate, pyruvate, fumarate, and glycerol as electron donors [57]. *A. oremlandii* grew only at pH 7.0 and in the absence of Cu–Zn-Pb-Ni–Cd (Fig. 3).

*S. turbinis* was previously isolated from shellfish [58], organisms widely present in Guanabara Bay [59]. According to the literature, this bacterium is moderately anaerobic and thermophilic, has an optimal growth temperature and pH of 60 °C and pH 8.0, does not ferment sugar, and has weak EST enzymatic activity, and the major fatty acid in the cell is C_16:0_ [58]. *S. turbinis* also predominated in the bioassay with Cu–Zn-Pb-Ni–Cd (Fig. 3). This species was isolated from areas contaminated by sanitary sewage and was grown in aerobic conditions using yeast extract and urea as carbon and energy sources. There are no references on the growth of this species in the presence of multiple metals.

*Rhodoplanes* spp. grow on the subsurface of sediments, are phototrophic and mobile, and have a great diversity of hopanoids and lipid biomarkers of biological activity in sedimentary rocks. The major cell hopanoids in *R. piscinae* JA266^T^ are diplopterol (V) and its methylated product 2-methyldiplopterol (VI) [60]. Hopanoids are widely used in the chemotaxonomy of bacteria [61]. This species was also isolated from the sediment of a bay with a history of contamination by sanitary and industrial sewage, and the current study provides the first evidence that *R. piscinae* also grows in the presence of multiple metals (Fig. 3).

Heavy metals such as Cu, Zn, Pb, Ni, and Cd are the most dangerous metals [62], and the Jurujuba Sound area, where the sediment samples were collected, has high concentrations of Zn, Cu, and Pb [10]. Sites impacted by heavy metals select for microorganisms with the ability to resist one or several metals by reducing their toxic effects through various mechanisms [63].

Although bacterial species fluctuated during the experiments, significant changes in richness and diversity between the control and treatment were not observed (Online Resource 21). Also, there was no observed prevalence of pathogenic bacteria and pH was an important factor in the maintenance and/or selection of species in the bioassays in the absence of metals. Neutral pH favored the maintenance of bacterial species for 11 days. At acidic pH, initially, two species predominated, and two other species were added at the end of the control bioassay. Most likely, *O. chironomi* and *T. creatinophila*, by metabolizing organic matter, created a physicochemical environment favorable to the growth of *H. miurensis* and *A. oremlandii* at the end of the experiment.

**Fig. 4:**
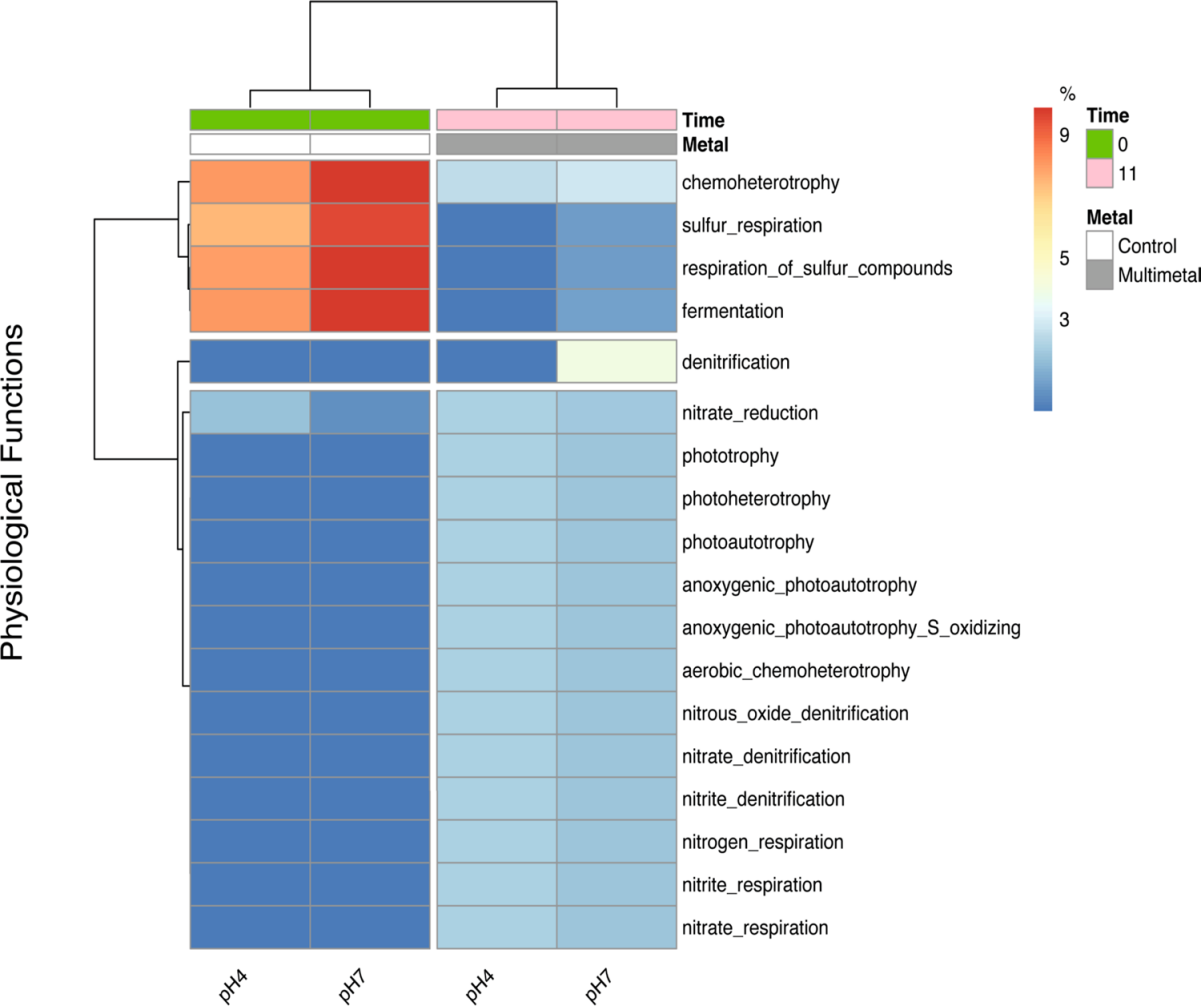
Heatmap of microbial physiological functions predicted by FAPROTAX from tested bacterial consortium between control and metal solution (Cu, Zn, Pb, Ni, and Cd, CONAMA Resolution 357/2005 limit), with pH 4 and 7. The graph was based on the relative abundance of bacterial taxa in experiment and nonmetric dimensional scaling (NMDS) analysis was used to show composition clustering according to the treatment

In the predictive metabolism results, the bacterial consortium in control showed lower potential physiological diversity based on the recovered pathways. FAPROTAX assigned 21.2% of bacterial taxa to at least one physiological function, due to lack of public domain information on cultivated marine microorganisms [64, 65] and limitations of FAPROTAX [28]. The most prevalent pathways in control are those related to sulfur, fermentation, and chemoheterotrophy (Fig. 4), mainly in pH 7. It indicated a microbial community that grows in low oxygen concentration while depleting nutrients from yeast extract of liquid medium.

In contrast, the pathways involving sulfur and fermentation were less common in the treatment groups, which tended to include chemoheterotrophy and metabolism involving the usage of nitrate and nitrite (Fig. 4). It suggests that the studied consortium shifts its metabolism to recycle nitrogenous compounds in the presence of metals. The data indicates the participation of microbes in the consumption of cellular components, especially proteins and nitrogenous organic compounds produced by bacteria that perished during the assay.

Microorganisms could be used in bioremediation to speed up metal remediation [14], due to their capacity to act as biosorbents or their production of secondary metabolic compounds that transform toxic heavy metals into less harmful products. In this context, polluted areas are a source of novel microbes that can be used to speed up remediation [17, 18]. Our data indicated that isolated consortium might endure extremely contaminated conditions and tolerate high multimetal concentrations. Guanabara Bay might have chosen microbial genetic traits that give tolerance to metal stress through time [20] to bacteria that compose isolated consortium. Bacteria grew in treatment groups with pH values, temperatures, and carbon and energy sources different from those mentioned in the literature, with low changes in diversity profile, no presence of pathogenic microorganisms, and a slight cell number decrease in T11, despite Cu–Zn-Pb-Ni–Cd inhibition on DHA and EST enzymes. These observations make sampled bacterial consortium a promising tool for multimetal bioremediation.

## Conclusion

In this study, bacterial consortia grew in treatment groups with pH values, temperatures, and carbon and energy sources different from those mentioned in the literature. The diversity profile changed little during the experiment, with a slight cell number decrease in T11. The change in diversity was small and did not affect the persistence of the consortium under Cu–Zn-Pb-Ni–Cd exposure. Regarding the potential physiology, the changes favored the maintenance of the group, with an emphasis on organisms capable of recycling compounds left by the bacteria that perished during the bioassay. The results are significant because they show no prevalence of pathogenic bacteria in the process. The microbial population can metabolize and precipitate Cu–Zn-Pb-Ni–Cd and is susceptible to ecological succession at the end of the bioassay, indicating that sampled bacterial consortium is a promising tool for multimetal bioremediation.

## Supplementary information

Below is the link to the electronic supplementary material.

Supplementary file1 (DOCX 4200 KB)

## Data Availability

The data generated and analyzed in the current study are presented within the manuscript and its supplementary data. Genomic data are deposited in NCBI (SUB12127057 and SAMN31169424) and with legal registration SISGEN A4A68B5.
