# Supplementary material for: Biomass and enzymatic activities of marine bacteria in the presence of multiple metals
Source: Braz J Microbiol. 2023 May 22;54(3):1523–32. doi: 10.1007/s42770-023-00993-5 (PMC10485232; doi:10.1007/s42770-023-00993-5)
Supplement: Supplementary file 1 — Supplementary file1 (DOCX 4200 KB) [file 42770_2023_993_MOESM1_ESM.docx]

*BIOMASS AND ENZYMATIC ACTIVITIES OF MARINE BACTERIA* *IN THE PRESENCE OF MULTIPLE METALS*

Bitencourt, JAP^1*^; Checker, LPT^2^; Waite, CC^2^; Oliveira, G.^2, 3^; Oliveira, AMS^1^, Pereira, DC^2^ & Crapez, MAC^2^

^1^ Instituto Tecnológico Vale, Belém, PA, CEP 66055-090, Brazil.

^2^ Departamento de Biologia Marinha, Programa de Pós-graduação em Biologia Marinha e Ambientes Costeiros, Universidade Federal Fluminense, Niterói, RJ, CEP 24020-150, Brazil.

^3^ School of Earth and Environmental Sciences, University of Queensland, St. Lucia, Brisbane, 4072, Queensland.

*Corresponding author. E-mail address: jose.augusto.bitencourt@itv.org. Tel.: +55 (91) 3213-5583

**OR. 1** Results of nested Analysis of variance (ANOVA) of growth, enzyme and biopolymeric production of tested bacterial consortium in the presence of metal solution (Cu, Zn, Pb, Ni and Cd) at concentrations of 0 (control) and environmental standard limit (CONAMA Resolution 357/2005) and adjusted pH (4 and 7) during 11 days (T0, T5 and T11). Significance is represented by p < 0.05 and bold marked, d.f. = degrees of freedom, SS = sum of squares, MS = mean of squares and F = f-statistic.

| Number of cells (cell.mL^-1^) | df | SS | MS | F | p-value |
| --- | --- | --- | --- | --- | --- |
| pH | 1 | 196.544 | 196.544 | 295.333 | 0.098 |
| pH:factor(Concentration) | 2 | 3.081 | 1.540 | 231.536 | **<0,01** |
| pH:factor(Concentration):factor(Time) | 8 | 66.091 | 826.137 | 124.137 | **<0,01** |
|  |  |  |  |  |  |
| Esterase (μg Fluorescein.h. mL^-1^) | df | SS | MS | F | p-value |
| pH | 1 | 21.777 | 21.777 | 115.040 | **0.002** |
| pH:factor(Concentration) | 2 | 358.937 | 179.468 | 948.038 | **<0.001** |
| pH:factor(Concentration):factor(Time) | 8 | 1.003 | 125.444 | 662.654 | **<0.001** |
|  |  |  |  |  |  |
| Dehydrogenase (µg O2.h.mL^-1^) | df | SS | MS | F | p-value |
| pH | 1 | 0.004 | 0.0040 | 389.707 | **0.024** |
| pH:factor(Concentration) | 2 | 0.01481 | 0.0074 | 71.030 | **<0.001** |
| pH:factor(Concentration):factor(Time) | 8 | 0.02377 | 0.0029 | 284.886 | **<0.001** |
|  |  |  |  |  |  |
| Carbohydrates | df | SS | MS | F | p-value |
| pH | 1 | 650.25 | 650.25 | 0.3288 | 0.572 |
| pH:factor(Concentration) | 2 | 2562.5 | 1281.25 | 0.6480 | 0.532 |
| pH:factor(Concentration):factor(Time) | 8 | 16308 | 2038.5 | 103.106 | 0.441 |
|  |  |  |  |  |  |
| Proteins | df | SS | MS | F | p-value |
| pH | 1 | 999.999 | 999.999 | 0.9069 | 0.350 |
| pH:factor(Concentration) | 2 | 150.895 | 754.475 | 684.254 | 0.441 |
| pH:factor(Concentration):factor(Time) | 8 | 331.419 | 41.427 | 375.717 | 0.560 |
|  |  |  |  |  |  |
| Lipids | df | SS | MS | F | p-value |
| pH | 1 | 460.460 | 460.460 | 710.582 | **0.014** |
| pH:factor(Concentration) | 2 | 324.118 | 162.059 | 25.009 | **0.008** |
| pH:factor(Concentration):factor(Time) | 8 | 616.979 | 771.223 | 119.015 | **0.002** |


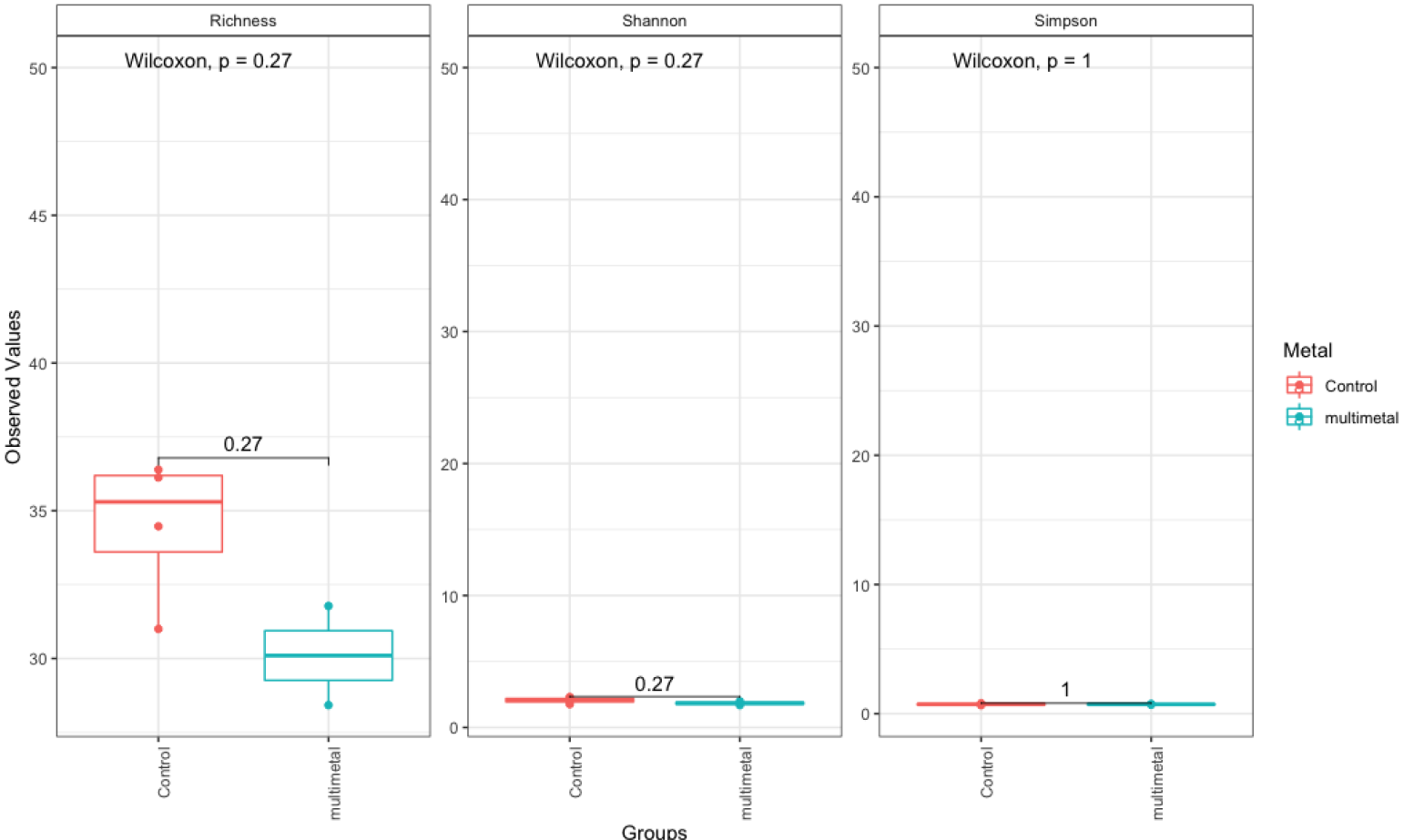


OR. 2 The differences in bacteria community diversity and richness indexes of tested bacterial consortium in the presence of metal solution (Cu, Zn, Pb, Ni and Cd) at concentrations of 0 (control) and environmental standard limit (CONAMA Resolution 357/2005, multimetal).
